# Supplementary material for: PI3K-AKT activation resculpts integrin signaling to drive filamentous tau-induced proinflammatory astrogliosis
Source: Res Sq. 2023 Aug 23:rs.3.rs-3253118. Preprint. [Version 1] doi: 10.21203/rs.3.rs-3253118/v1 (PMC10479431; doi:10.21203/rs.3.rs-3253118/v1)
Supplement: Supplement 1 [file NIHPPrs3253118v1-supplement-1.pdf]

Supplementary Files

This is a list of supplementary files associated with this preprint. Click to download.

- SupplementaryInformation.docx
- TableS1.xlsx
- TableS2.xlsx
- TableS3.xlsx
- TableS4.xlsx
- TableS5.xls
- TableS6.xlsx
